# Reviews and Book Notices

**Published:** 1878-06

**Authors:** 


### Reviews and Book Notices.

---

The Advantages and Accidents of Artificial Anæsthesia. Being a manual of anæsthetic agents, and their modes of administration, considering their relative risk, tests of purity, treatment of asphyxia, etc. By Laurence Turnbull, M. D., Ph. G., etc. Philadelphia: Lindsay & Blakiston, 1878. pp. 210.

Whenever the question of anæsthesia and the relative safety of the different anæsthetics is ventilated by a body of medical men, the desultory manner of the discussion is its most noteworthy feature. The earnestness with which the subject is discussed, and the great number participating actively in the debate, show the profound interest medical men take in this all-important question. While, on the other hand, the opinions of the different speakers, generally founded solely upon their limited personal experience, betray the fact that physicians are often ignorant of a great many things which have been published in relation to this subject of anæsthesia.

We do not make these remarks for the purpose of casting any reproach upon our professional friends; we simply wish to point to the fact that valuable information which is annually published in hundreds of medical periodicals in the different tongues of the civilized world, is practically inaccessible to the busy practitioner. A book, therefore, which presents this information in the condensed form of a small compendium, must be welcome to every physician, for it meets a great real want. A good compilation of this kind represents a vast amount of work, and also reflects the sound judgment of the author in critically sifting the material.

Such is the character of the little book before us. In a concise form, the properties and actions of the agents now used as anæsthetics are exhibited; the best methods of their administration are well described, and directions added for prompt action in cases of asphyxia and other accidents. The question as to the comparative safety of the different anæsthetics (especially ether and coloroform), is thoroughly ventilated and impartially decided in favor of sulphuric ether.

If suggestions are admissible, we would remark that among the secondary effects of ether, the occasional occurrence of neuralgic affections should have been mentioned. The fact that neuralgia sometimes follows the inhalation of ether, seldom though it may happen, is of sufficient practical importance to deserve a place in a complete treatise on anæsthetics.

This excellent little work cannot be too warmly recommended to every busy practitioner who wishes to obtain much information by little reading. F. C. H.

Modern Organic Chemistry. By C. Gilbert Wheeler, Prof. of Chemistry in the University of Chicago. Chicago: Jansen, McClurg & Co.

The need of a good text book, devoted entirely to organic chemistry, has long been felt, both by teachers and students. The subject is one of such great extent, and of such practical importance, from its fruitful relations to the arts and to medicine, that the brief chapters usually devoted to it in works on general chemistry, are thoroughly insufficient for the needs of the conscientious student, while at the same time the elaborate volumes of Miller, Wöhler and others are too extended for general use. Prof. Wheeler's new work admirably fills the void, placing in our hands a clear outline of modern organic chemistry, at once compendious and yet giving an ample exposition of all important parts of the subject.

Considerable attention has been given to the medical relations of the different subjects treated of, giving the work an additional value to the student and practitioner of medicine. The chapter on the alkaloids is particularly full and will well repay careful study; for, as a general rule, far too little is known by medical men of the chemical relations of these highly important remedial and toxic agents. Claude Bernard's valuable table of the relative toxic, convulsive and soporific value of the different alkaloids in opium is reproduced, and is well worthy of careful attention and remembrance. The alcohols, with their numerous derivatives, many of which are put to such extended use in medicine, receive careful treatment, while the organic acids and bases are discussed clearly and at sufficient length.

We are sorry to see an occasional rather loose use of chemical terms, such as soda and potassa, where evidently sodic and potassic hydrates are referred to—of the ambiguous term hyponitric acid, etc. The subject of chemical nomenclature is in a state of such confusion, that unless one is careful to use exclusively either the old or the new system, the greatest perplexity may arise, especially in the minds of the beginner. While in conversation, or even in the lecture room, an occasional employment of the loose, old-fashioned names of chemicals may be quite admissible, yet in a text-book designed for accurate scientific training, their use can scarcely be too severely reprehended.

Typographically, the work reflects the very greatest credit upon its western publisher and printer. The impression is beautifully clear and distinct, the paper thick and softly tinted, the arrangement of the various tables admirable, while the almost unexceptionable accuracy with which the complex formulæ of organic substances is given, challenges our highest admiration.

W. S. H.

FORENSIC MEDICINE AND TOXICOLOGY. By W. Bathurst Woodman, M.D., F. R. C. P., and Charles Meymott Tidy, M.B., F. C. S., Philadelphia: Lindsay & Blackiston. 8vo.

We have examined this work with interest and find little in it to blame, while there is much that is worthy of praise. It forms a volume of 1,054 pages, of which about one half is devoted to Toxicology.

The first chapter gives the chief practical issues which arise for decision by medical witnesses, and then tells what principles should guide them in giving evidence in the courts.

Chapter second—comprising only four pages and a half—is an excellent one upon the examination of bodies found dead, and gives full directions how to make the proper *post mortem*. The third chapter discusses very thoroughly " Signs of Death," and mentions that the Paris " Academy of Sciences," in 1873, offered a principal prize of 20,000 frs. " for the discovery of a simple and popular mode of recognizing the signs of real death in a certain and indubitable manner, a method which may be put in practice by poor uneducated villagers." Sixteen very practical pages are given to this subject.

Any physician reading these three chapters will more clearly apprehend the scope of this branch of medical science, and in the new interest and appreciation awakened in him will find ample recompense for his trouble.

Chapters fourth to eighteenth, inclusive, are devoted to Toxicology, and after careful examination we feel justified in saying that nothing has yet been written which gives so much information upon the subject and that in so clear and condensed a manner. In examining a poison the method of our author is to give:

1. The description of the substance.
2. Its different preparations.
3. Results of experiments on animals.
4. Dose, and symptoms of poisoning by it.
5. Treatment.
6. *Post mortem* appearances.
7. Tests.
8. Toxicological analysis.
9. Quantitative estimation.
10. A Table of cases of poisoning by the substance indicated.

This table is very valuable and gives in each case:

(a.) The source from which it is quoted, the particular form of the poison taken and the amount.

(b.) Symptoms.

(c.) Result.

(d.) *Post mortem* (if any).

One hundred and twenty-one cases of opium poisoning are thus given, eighty-nine of strychnia, and even under the head of poisoning by mushrooms we have fourteen cases. Other rare poisons are treated in the same careful manner, making the work, in this particular, highly satisfactory. It is gratifying to us, as

Americans, to find such liberal quotations from Wormley and other writers upon Toxicology in our own land.

Chapter nineteenth treats of the examination of hairs and stains. It teaches very effectively the employment of the microscope, spectroscope and chemistry for this purpose.

Chapter twentieth is upon life insurance, and contains a table of cases of disputed policies, with the medico-legal questions involved, and a list of some causes of sudden death.

Chapter twenty-first gives thirty pages upon "Personal Identity," and discusses, in a masterly manner, this subject—often a most troublesome one to the medical jurist.

The latter part of the book seems to us less carefully prepared than the preceding portions. There is valuable matter in each chapter, but it is neither well digested nor well arranged. For example: the subject of "Sexual Relations" is treated in chapters twenty second and twenty-fifth, and quite imperfectly, while chapter twenty-fourth, upon "Malapraxis," is very strangely thrown in between the twentieth, upon "Pregnancy," and the twenty-sixth, upon "Premature Labor," which would naturally be expected to come together and to be immediately followed by those upon the "Sexual Relations"; and the subject of "Malapraxis" should follow that of "Wounds and Injuries."

The comparatively short space of one chapter is allotted to "Mental Unsoundness," and, although much valuable information is there given in a very condensed form, it is necessarily far from sufficient to satisfy physicians upon this most perplexing of all medico-legal subjects.

The same complaint may be made in regard to "Wounds and Injuries," to which but one chapter, the last, is devoted, whereas the subject is one of such undoubted and generally conceded importance, that a work of so high a character as that in hand should not have passed it by without an extended exposition.

As a whole, despite the defects to which we have considered it the duty of impartial criticism to call attention, we pronounce the volume before us an important addition to our medical literature, and cordially recommend it to the careful perusal of our readers.

Clear type, broad margins and tasteful binding give the book an attractive appearance and bring it fully up to that high standard of typographical excellence established by Lindsay & Blackiston. H. P. M.

TRANSACTIONS OF THE AMERICAN DERMATOLOGICAL ASSOCIATION, with the President's Address, at the First Meeting held at Niagara, Sept. 4, 5 and 6, 1877. N. Y.: G. P. Putnam's Sons.

This little pamphlet of 42 pages illustrates a new departure from the traditional procedure of scientific associations. Instead of publishing a large and expensive volume, which would have been read by few except those especially interested in such literature, the American Dermatological Association decided to permit its members to furnish their papers for publication to the various Medical Journals throughout the country. The result has been that these journals have been enriched during the year past with many valuable and important papers (two of them have appeared in the CHICAGO MEDICAL JOURNAL AND EXAMINER); and these papers have also been placed in the hands of many readers who would doubtless otherwise have never had the opportunity of perusing them.

As a consequence, the real transactions of the Association are contained in the present pamphlet, including the President's address, which furnishes an admirable sketch of the progress of dermatological studies in America. This last is enriched by a tabulated list of contributions to dermatological literature in this country, arranged under the names of authors; the list including the names of White and Wigglesworth of Boston; Duhring and Van Harlingen, of Philadelphia; Keyes, Otis, Bulkley, Piffard, Taylor and Bumstead of New York, and others whose writings are familiar to the profession in the United States.

We are inclined to believe that this method of utilizing the work done by societies devoted to the advancement of any special department of medical science, will meet with the approval of the profession at large. J. N. H.

